# Improving the Weak Gel Structure of an Oil-Based Drilling Fluid by Using a Polyamide Wax

**DOI:** 10.3390/gels8100631

**Published:** 2022-10-06

**Authors:** Xianbin Huang, Xu Meng, Mao Li, Jinsheng Sun, Kaihe Lv, Chongyang Gao

**Affiliations:** 1 Key Laboratory of Unconventional Oil & Gas Development, China University of Petroleum, East China, Ministry of Education, Qingdao 266580, China; 2 School of Petroleum Engineering, China University of Petroleum, East China, Qingdao 266580, China

**Keywords:** rheology modifier, oil-based drilling fluid, polyamide wax, weak gel

## Abstract

Oil-based drilling fluids (OBDFs) are widely used, but there are common problems associated with them, such as low yield point and poor cutting–carrying and hole cleaning ability. In this paper, a polyamide wax (TQ-1) was synthesized from dimeric acid and 1,6-hexanediamine to improve the weak gel structure of OBDFs. The TQ-1 was characterized by Fourier transform infrared spectroscopy (FTIR) and thermogravimetric analysis (TGA). Then the effect of the TQ-1 on the stability of the water-in-oil emulsion was studied by sedimentation observation, stability analysis, an electrical stability test, and particle size measurement. The effect of the TQ-1 on the rheological properties of the water-in-oil emulsion was analyzed by viscosity vs. shear rate test and the three-interval thixotropic test. Finally, the performance of the TQ-1 in OBDFs was comprehensively evaluated. The experimental results showed that the initial thermal decomposition temperature of the TQ-1 was 195 °C, indicating that the TQ-1 had good thermal stability. After adding the TQ-1, the emulsion became more stable since the emulsion stability index (TSI) value decreased when the emulsions were placed for a period of time and the demulsification voltage was increased. The TQ-1 could form a weak gel structure in the water-in-oil emulsions, which made the emulsions show excellent shear thinning and thixotropy. TQ-1 can improve the demulsification voltage of OBDFs, greatly improve the yield point and gel strength, and largely reduce the sedimentation factor (SF). In addition, TQ-1 has good compatibility with OBDFs, and in our study the high-temperature and high-pressure (HTHP) filtration decreased slightly after adding the TQ-1. According to theoretical analysis, the mechanism of TQ-1 of improving the weak gel structure of OBDFs is that the polar amide group can form a spatial network structure in nonpolar solvents through hydrogen bonding.

## 1. Introduction

Drilling fluids are facing increasing technical challenges as the development of oil and gas resources extends from conventional to unconventional and deep oil and gas [1]. Oil-based drilling fluids (OBDFs, the lists of abbreviations are provided in Appendix A Table A1) have favorable lubrication, high-temperature resistance, anti-collapse, and contamination resistance performance [2,3], playing a vital role in the development of shale oil and gas and in deep reservoirs [4]. Although OBDFs have unique advantages in horizontal and deep wells [5], the low yield point and gel strength of high-density OBDFs under high-temperature conditions affect the ability to carry cuttings and suspend weighting materials [6]. 

Drilling fluids are expected to have a weak gel structure [7,8,9,10]. At a low shear rate, the viscosity is high, which is beneficial to suspend drill cuttings; at a high shear rate, the viscosity is low, which is conducive to fast drilling. At present, organoclay [11] is the predominant method used worldwide to improve the rheology of OBDFs [12,13]. Although organoclay can improve the rheology of drilling fluid, it reduces the solid phase capacity of the fluid and affects the rheology of high-density drilling fluid in particular, resulting in a negative impact on the rate of penetration [14,15]. Organoclay cannot fully meet the rheology requirements of OBDFs under high-density conditions [6]. Therefore, worldwide research has been conducted on rheology modifiers for OBDFs. Currently, rheology modifiers for OBDFs include oil-soluble polymers [16,17], modified fatty acids [18], and nanocomposites [19,20]. Ma et al. [16] synthesized a new oil-soluble polymer by suspension polymerization using methyl styrene, methyl methacrylate, stearyl acrylate, and dimethyl 2,2 azobis isobutyrate as raw materials, which effectively improved the yield point of OBDFs under 180 °C conditions. Shi et al. [7] synthesized a dendritic polymer with dodecyl dibasic acid and trientine as the raw materials, which effectively improved the rheology of the OBDFs and the dispersion of organoclay in the oil. However, polymer-based rheology modifiers have a significant influence on the viscosity of drilling fluids, and this situation is not conducive to the regulation of rheology under high-density conditions. In our previous study [18], the reaction product of ethylene glycol amine with dimer acid was used as a rheology modifier, which greatly enhanced the zero-shear-rate viscosity of the water-in-oil emulsion. Noah et al. [20] prepared a nanocomposite rheology modifier by adding nano-zinc oxide (ZnO-NPs) and nano-calcium carbonate (nano-CaCO_3_) to a polystyrene-butadiene rubber (PSBR) copolymer, which effectively improved the yield point (YP), apparent viscosity (AV), and plastic viscosity (PV) of the OBDFs. Madkour et al. [21] enhanced the suspension performance of drilling fluids by synthesizing a biodegradable nanocomposite as a rheology modifier using solution casting techniques. Since rheology modifiers for OBDFs have the effect of improving the yield point and suspension stability, at present the performance is limited under high-temperature and high-density conditions. 

From an in-depth study of the mechanisms involved in regulating rheology in OBDFs, Sun et al. [15] and Ma et al. [16] discovered that the strong polar groups of the rheology modifiers formed a network structure by interacting with other molecular groups through hydrogen bonds or electrostatic forces. This structure further enhanced the weak gel structure in the drilling fluid, thereby improving the yield point and suspension stability of the drilling fluid.

To improve the rheological properties of OBDFs, in this work a polyamide wax (TQ-1) was developed that could effectively enhance the weak gel structure of the drilling fluids. In addition, the TQ-1 was characterized by Fourier transform infrared spectroscopy (FTIR) and thermogravimetry analysis (TGA). The effects of TQ-1 on emulsion stability and rheology were studied, and the performance of TQ-1 in OBDFs was evaluated. Our results showed that TQ-1 could increase the yield point and gel strength but had little effect on the plastic viscosity.

## 2. Results and Discussion

### 2.1. FTIR

**Figure 1:**
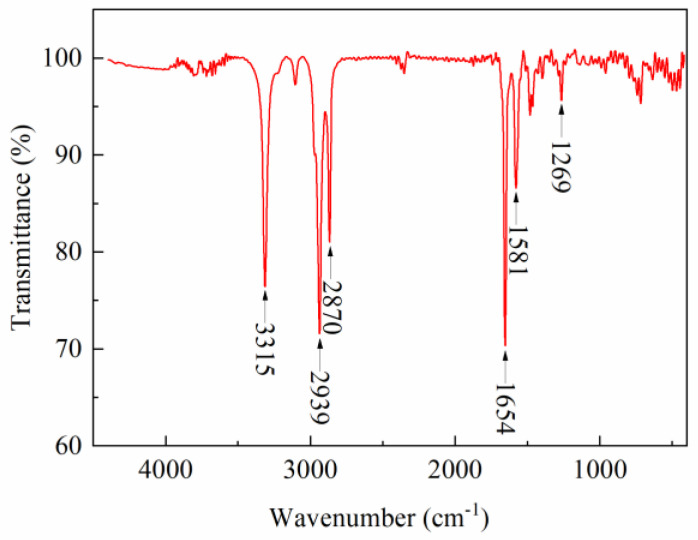
Infrared spectra of TQ-1.

Figure 1 shows the infrared spectrum of TQ-1, where the absorption peak at 3315 cm^−1^ was the N-H stretching vibration peak in 1,6 ethylenediamine, the CH_2_ stretching vibration peaks for dimeric acid were at 2939 cm^−1^ and 2870 cm^−1^, the C=O stretching vibration peak of dimeric acid was at 1654 cm^−1^, and the peak at 1581 cm^−1^ corresponded to the N-H bending vibration peak of 1,6 ethylenediamine. In addition, the peak at 1269 cm^−1^ corresponded to the C-N stretching vibration peak of 1,6 ethylenediamine. Evidently, the synthesized TQ-1 molecules contained amide and long-chain alkyl groups, which indicated that the molecular structure of TQ-1 conforms to the design.

### 2.2. TGA

**Figure 2:**
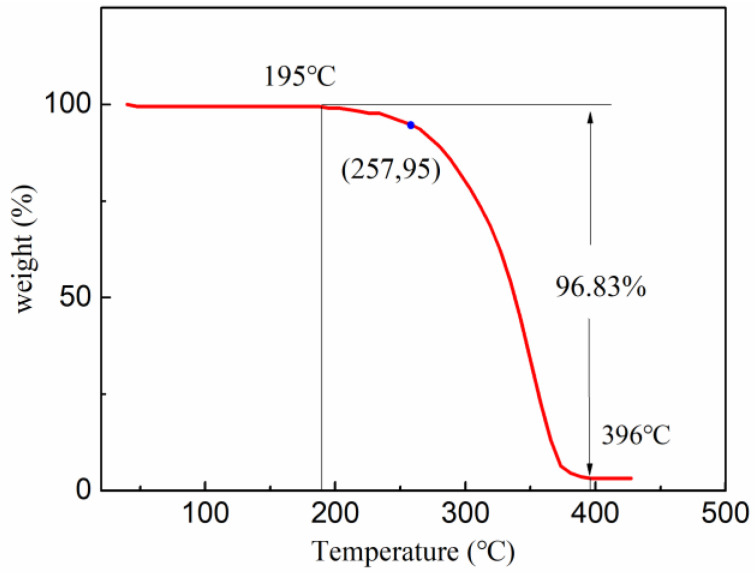
Thermogravimetric curve of TQ-1.

Figure 2 shows the thermogravimetric curve of TQ-1, which could be roughly divided into three stages. In the first stage, before 195 °C, there was no change in the weight of the sample. In the second stage, between 195 °C and 396 °C, the weight of the sample degraded, slowly at first and then rapidly. The slow weight loss was mainly due to the volatilization of the partial unreacted monomers and the decomposition of a small number of side chains. The rapid weight loss after about 257 °C was caused by thorough decomposition of both side and main chains. In the third stage, after 396 °C, there was essentially no loss in the weight of the TQ-1. Overall, the initial decomposition temperature of the TQ-1 was 195 °C and the weight loss was only 5% at 257 °C, which showed that TQ-1 had favorable resistance to high temperatures.

### 2.3. Emulsion Stability

#### 2.3.1. Sedimentation Observation

**Figure 3:**
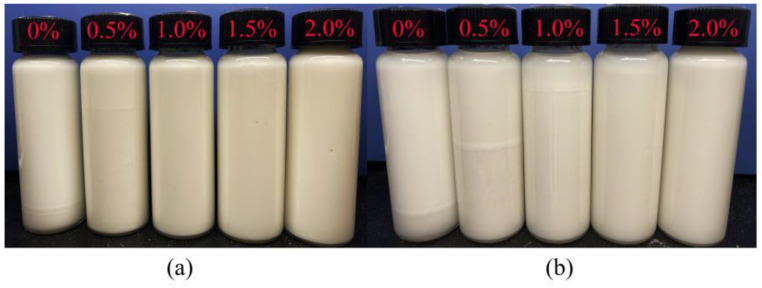
Sedimentation stability of the invert emulsions with different TQ-1 concentrations set for 1 (**a**) and 7 (**b**) days.

The effect of TQ-1 on the stability of the invert emulsions with an oil-to-water ratio of 80:20 was studied by the observation method, and the experimental results are shown in Figure 3. Severe sedimentation occurred after 24 h and 7 d for emulsions without TQ-1. With higher concentrations of TQ-1, the volume of the oil that precipitated in the upper layer gradually decreased and the sedimentation phenomenon weakened (Figure 3a). No sedimentation was observed for emulsions with 1.0%, 1.5%, and 2.0% TQ-1 concentrations after 24 h. When the standing time was extended to 7 days, the emulsion with a TQ-1 concentration of 1.0% had slight sedimentation (Figure 3b). However, with increased TQ-1 concentrations of 1.5% and 2.0%, the stability remained favorable after a standing time of 7 days. Therefore, TQ-1 effectively enhanced the sedimentation stability of the emulsions. Moreover, the ability to stabilize the emulsion became more pronounced as the concentration of TQ-1 increased.

#### 2.3.2. Emulsion Stability Index (TSI)

**Figure 4:**
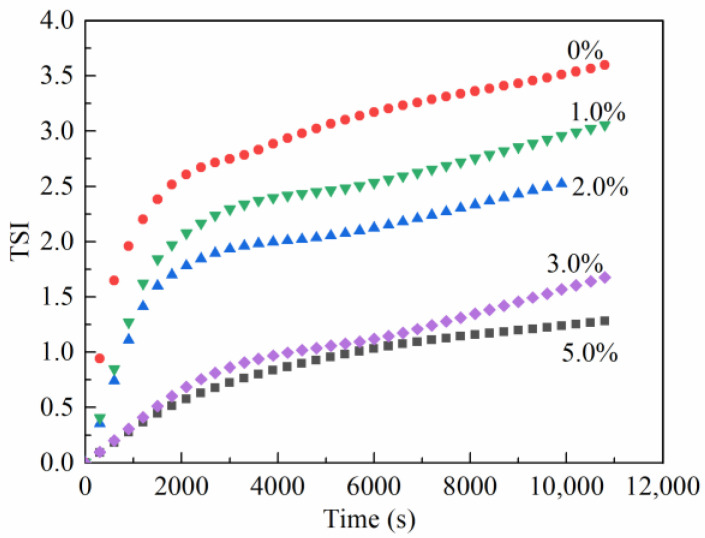
Effect of TQ-1 on the TSI values of the invert emulsions.

The emulsion stability index [22,23] can monitor unstable kinetics in samples over time and is a Turbiscan^®^-specific parameter developed for formulators to compare and characterize the physical stability of various formulations. In this study, the TSI values for invert emulsions with different concentrations of TQ-1 were obtained by a stability tester, and the experimental results are shown in Figure 4. For all the emulsion samples, the TSI values gradually increased over time, indicating that the emulsion has a tendency to become unstable with time. In the same scan, the TSI values decreased as the concentration of TQ-1 increased. Therefore, the addition of TQ-1 improved the stability of the emulsion.

#### 2.3.3. Electrical Stability (ES) Test

**Figure 5:**
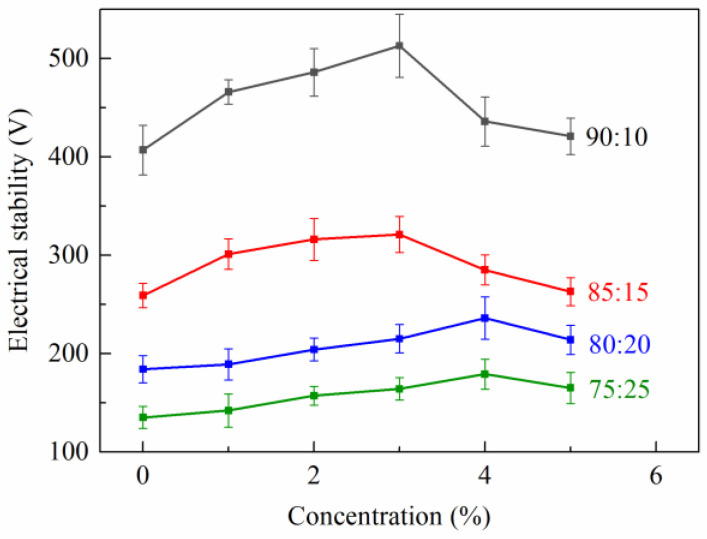
Effect of TQ-1 on the electrical stability of emulsions with different oil-to-water ratios (error bars represent standard deviation). The standard deviation values range from 9.5 to 32.0 V based on the oil-to-water ratio and TQ-1 concentration.

The demulsification voltage is a key parameter used to measure the strength of emulsion stability. As shown in Figure 5, for the emulsions with different oil-to-water ratios, the addition of TQ-1 corresponded to an increasing demulsification voltage in the sample. When the oil-to-water ratio was 90:10, the demulsification voltage of the emulsion ranged from 407 to 513 V when the TQ-1 concentrations increased from 0% to 3.0%. However, with the further increase in TQ-1 concentration, the demulsification voltage first increased then decreased. This was due to the formation of a stable network structure after the TQ-1 was added, which played an auxiliary stabilizing role in electrical stability. When the concentration of TQ-1 was low, an increase in TQ-1 resulted in the gradual strengthening of the network structure and electrical stability. However, as the quantity of TQ-1 continued to increase, the network structure tended toward saturation, and the strongly polarized amide groups in the TQ-1 molecule were directly adsorbed on the water surface, thereby destroying the stability of the invert emulsion and worsening the electrical stability. In summary, TQ-1 could improve the electrical stability of the invert emulsion at a proper concentration. 

#### 2.3.4. Particle Size Analysis of the Emulsion

**Figure 6:**
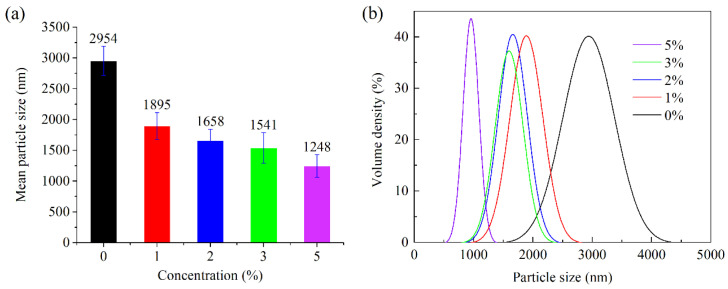
Effect of different concentrations of TQ-1 on the mean particle size of the emulsion. (**a**) mean particle size (error bars represent standard deviation), with the standard deviation values ranging from 182 to 247 nm based on the TQ-1 concentration; (**b**) particle size distribution curves.

The prepared samples were set for 8 h, and the particle size of the emulsions was determined by a focused beam reflectance measurement (FBRM) instrument. As shown in Figure 6, the samples with TQ-1 had a smaller mean particle size compared to those without TQ-1. As the concentration of TQ-1 increased, the peak in the particle size distribution of the emulsion gradually shifted left toward smaller particle sizes. This was possibly due to the fact that the addition of TQ-1 gradually stabilized the network structure, thus improving the stability of the entire system and slowing the aggregation of the internal phases of the emulsion. Therefore, the particle size test also showed that TQ-1 contributed to the stability of the emulsion.

### 2.4. Rheological Performance

#### 2.4.1. The Effect of TQ-1 on the Viscosity of the Emulsion

**Figure 7:**
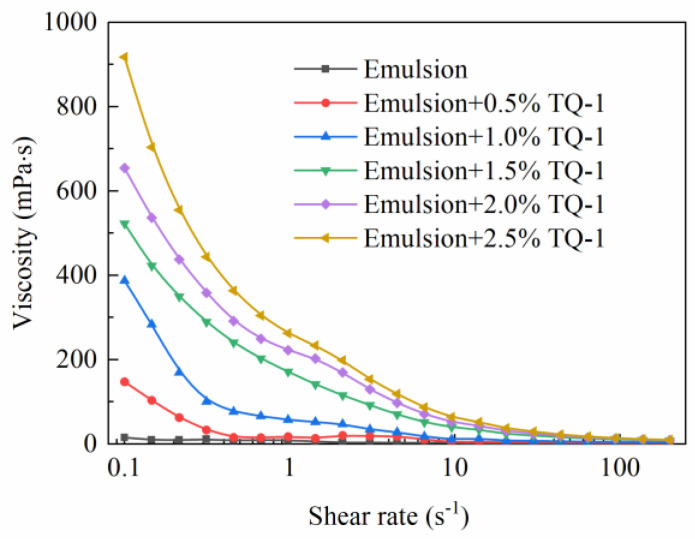
Effect of TQ-1 on the rheological curve of the invert emulsion.

Figure 7 shows the effect of TQ-1 on the rheology of the invert emulsion. When the shear rate was low, the viscosity of the emulsion increased substantially compared with the blank sample without TQ-1. At a shear rate of 0.1 s^−1^, the viscosity increased from 15.75 to 918 mPa·s when the concentration of TQ-1 increased from 0% to 2.5%. As the shear rate increased, the viscosity of the emulsion gradually decreased. When the shear rate increased from 0.1 to 100 s^−1^, there was little difference in the viscosity of the emulsion with and without TQ-1. This was due to the fact that a weak gel structure, which had a network structure with a stronger intensity at lower shear rates, formed in the emulsion after the TQ-1 was added. During the drilling process, this structure would facilitate a good carrying capacity for drill cuttings and the suspension of weighting materials when the circulation of the drilling fluid was stopped. When the shear rate was high, the weak gel structure was easily broken, which was conducive to reduction in drilling fluid resistance. The results showed that TQ-1 could effectively improve the rheological properties and enhance the shear thinning performance of the emulsion. 

#### 2.4.2. The Effect of TQ-1 on the Thixotropy of the Emulsion

**Figure 8:**
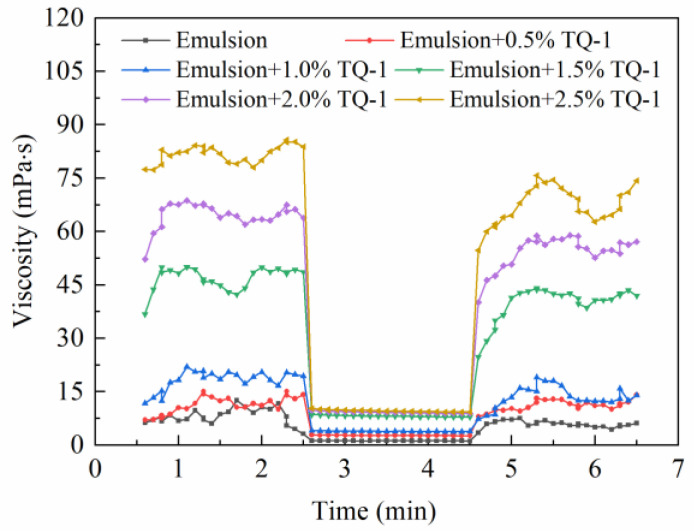
Three interval thixotropic curves of the invert emulsions under different TQ-1 concentrations.

As shown in Figure 8, the viscosity of the emulsion was relatively high under conditions of low shear rates. After adjustment to higher shear rates, the viscosity of the emulsion gradually declined and the differences in the viscosity among emulsions with different TQ-1 concentrations decreased. After returning to a low shear rate, the viscosity of the emulsion gradually recovered. This was due to the fact that the shear force affected the adsorption balance of the TQ-1, and under stronger shear forces, the network structure was broken and the viscosity was low. When the shear force was low, the network structure was re-established and the gel structure was restored. The drilling fluids are required to have good thixotropy [24]. When the drilling fluid circulation is stopped, the drilling fluid will have a certain yield point to facilitate the suspension of drill cuttings. When the cycle is resumed, the viscosity of the emulsion will rapidly decrease, and the pump pressure change will be low, which is not likely to cause pressure surging and complex accidents underground. The experimental results showed that after the addition of TQ-1, the emulsion had excellent thixotropy, which could meet the engineering needs of drilling fluids. 

### 2.5. The Effect of TQ-1 on the Performance of OBDFs

Rheological tests, electrical stability, and HTHP filtration loss experiments were used to evaluate the performance of the three drilling fluids (#1, #2, and #3) with a density of 2.0 g/cm^3^. The experimental results are shown in Table 1.

Compared to OBDFs without TQ-1, the drilling fluid with TQ-1 had a higher yield point and YP/PV ratio after aging. The magnitude of the yield point can be related to the ability of the drilling fluid to carry drill cuttings. Higher yield points will be capable of carrying drill cuttings at smaller annular velocities, and gel strength reflects the strength of the internal network structure of the drilling fluid. After the addition of TQ-1, the drilling fluid obtained a high yield point and gel strength, which indicated that the performance of the drilling fluid to carry the drill cuttings and its own settlement stability were greatly improved.

Drilling fluid with different concentrations of TQ-1 can be applied to different sections of the well. Formulation #2 met the required low yield point for the small borehole section, which could effectively avoid the phenomenon of excessive surge pressures. Formulation #3 met the requirements for horizontal, directional, and large displacement wells with high yield point and could effectively suspend drill cuttings. In addition, TQ-1 could slightly reduce the HTHP filtration loss of OBDFs. 

**Figure 9:**
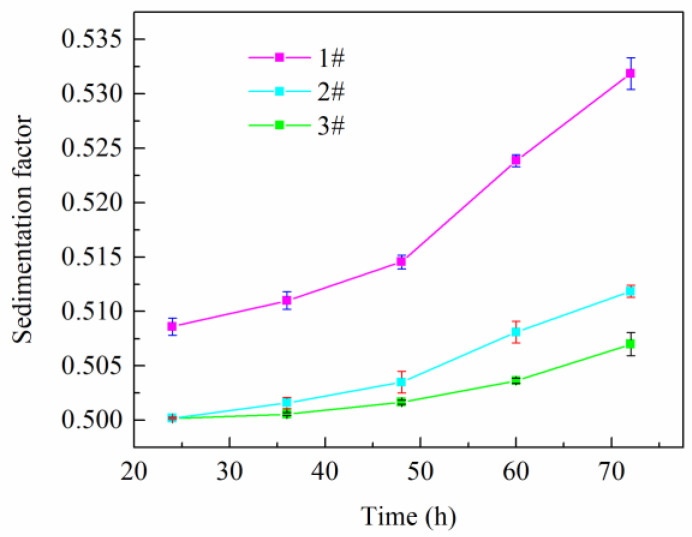
Changes in the sedimentation factors of different OBDFs over time (error bars represent standard deviation). The standard deviation values range from 9.4 × 10^−5^ to 1.5 × 10^−3^ based on TQ-1 concentration and sedimentation time.

By measuring the density values of the upper and lower layers of the drilling fluid, the sedimentation factors of the three drilling fluids after different standing periods were calculated and the experimental results are shown in Figure 9. As the standing time increased, the sedimentation factors of the three drilling fluids gradually increased. For Formulation #1, without TQ-1, the sedimentation factor was less than 0.52 after standing for 48 h and > 0.52 after standing for 60 h. Within three days, the sedimentation factors in Formulations #2 and #3 remained < 0.52, and Formulation #3 with the highest TQ-1 concentration had the smallest sedimentation factor, which after 72 h was only 0.507. The experimental results showed that TQ-1 could effectively enhance the sedimentation stability of OBDFs. 

### 2.6. Mechanism Analysis

**Figure 10:**
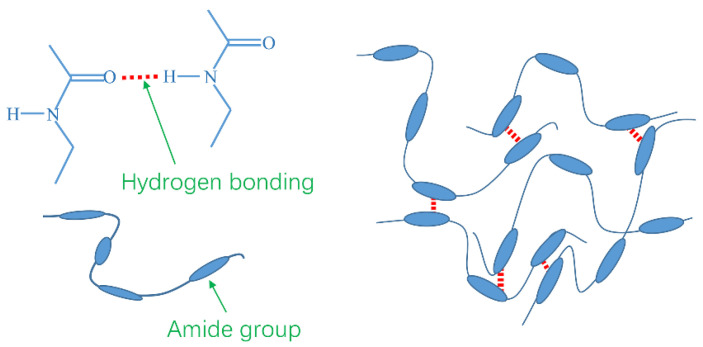
The schematic diagram of TQ-1 on improving the weak gel structure of oil-based drilling fluids.

The schematic diagram of TQ-1 on improving the weak gel structure of oil-based drilling fluids is shown in Figure 10. The chemical structure of the polyamide wax TQ-1 was mainly composed of polar amide groups and nonpolar aliphatic hydrocarbons. The oxygen atoms in the amide groups were electronegative, and hydrogen bonds between oxygen atoms and hydrogen atoms were easily formed. This type of hydrogen bond could form between different molecules or within the same molecule. The hydrogen bonds that formed within the same TQ-1 molecule caused curling in the molecular morphology, rendering it impossible to establish a spatial structure between the molecules. Different TQ-1 molecules could form a spatial network structure in a nonpolar solvent through hydrogen bonds, thus playing a role in improving the weak gel structure.

Under static conditions, a weak gel structure formed by TQ-1 favors suspended barite and drill cuttings and avoids stuck pipe, and this structure has a certain sensitivity to the shear rate. Under shear action, the breaking and formation of hydrogen bonds were in dynamic equilibrium and the hydrogen bonds were in a dynamic self-assembly state. When the shear rate was low, the spatial network structure of the OBDF was stronger, and when the shear rate increased, the spatial network structure became weaker and the viscosity of the drilling fluid decreased, which is beneficial to the rate of penetration. When the shear rate decreased again, the spatial network structure became stronger. Therefore, the OBDFs exhibited a certain thixotropy.

## 3. Conclusions

(1) A polyamide wax TQ-1 for OBDFs was synthesized by dimeric acid and 1,6-hexanediamine, with an initial decomposition temperature of 195 °C and good thermal stability.
(2) After adding TQ-1, both the sedimentation and emulsification stability of the emulsion are greatly improved. For emulsions with different oil–water ratios, TQ-1 can enhance the electrical stability of emulsion to different degrees.
(3) TQ-1 could effectively improve the rheological properties and enhance the shear thinning performance of the emulsion.
(4) TQ-1 increases the demulsification voltage, yield point, and gel strength and reduces settlement of OBDFs.
(5) TQ-1 mainly forms hydrogen bonds through polar amide groups, thus forming a spatial network structure to enhance the weak gel structure of OBDFs.

## 4. Materials and Methods

### 4.1. Materials

The dimeric acid (95 wt%) was purchased from Shanghai Maclean Biochemical Technology, Shanghai, China, and the 1,6-hexanediamide was purchased from Shanghai Aladdin Biochemical Technology Co. Ltd., Shanghai, China. The Span80 (98 wt%), calcium oxide (96 wt%), and anhydrous calcium chloride (96 wt%) were purchased from Sinopharm Chemical Reagent Co., Ltd., Shanghai, China, and the #5 white oil was obtained from Shenzhen ZRT Chemical Industry, Shenzhen, China. The industrial-grade organoclay, blown asphalt, and barite were obtained from Hubei Hanc New-Technology Co., Ltd., Jingzhou, China. The main emulsifier (modified fatty acid) and secondary emulsifier (fatty acid polyamine) were prepared in the laboratory. 

### 4.2. Preparation of TQ-1

The dimeric acid and 1,6-hexanediamine were successively placed into a round-bottom four-neck flask equipped with a stirring device, a water separator, and a condensing tube, where the stirring speed was set to 300 r/min. The oil bath temperature was set to 60 °C, and when the raw materials in the flask were completely dissolved, the oil bath temperature was adjusted to 195 °C. When the liquid temperature in the flask reached 195 °C, the reaction was heated at a constant temperature under a nitrogen environment for 3 h. When the reaction no longer generated water, the decompression reaction device was replaced and the reaction was decompressed for 2 h. After the end of the reaction, nitrogen was continuously passed until the reaction product was cooled to room temperature, completing the preparation of the polyamide wax TQ-1.

### 4.3. Characterization

#### 4.3.1. FTIR

TQ-1 was ground with KBr, mixed well, and pressed into sheets. The Fourier transform infrared (FTIR) absorption spectrum of the TQ-1 was measured in the range of 4000–400 cm^−1^ using an FTIR spectrometer (IRTRacer-100 type, Shimadzu, Japan).

#### 4.3.2. TGA

The thermogravimetric curve of the TQ-1 was tested in the range of 40 °C to 500 °C using a thermogravimetric analyzer (TGA2, Mettler Toledo, Greifensee, Switzerland) at a rate of 15 °C/min.

### 4.4. Emulsion Stability Tests

#### 4.4.1. Preparation of the Emulsion

First, 80 mL of white oil and 3 g of Span80 were added to each of five 150 mL beakers and stirred at 500 rpm for 2 h until the Span80 was sufficiently dissolved. 0, 0.5, 1.0, 1.5, and 2.0 g TQ-1 were added to each of the five beakers, respectively, and stirred for another 2 h, after which 20 mL of deionized water was added to each beaker. The emulsions with different TQ-1 concentrations were obtained using a high-speed shear emulsification mixer at 2000 rpm.

#### 4.4.2. Emulsion Stability

The effect of TQ-1 on emulsion stability was studied in the following four ways. First, 10 mL of the emulsions containing the different concentrations of TQ-1 were put into transparent vials and left to stand at room temperature for 1 and 7 days to observe the sedimentation of the emulsion.

Second, the emulsion stability was analyzed using a TURBISCAN LAB stability analyzer (Formulaction, Paris, France). The scanning time was set to every 10 min for 3 h, and the test temperature was set to 30 °C. The stability index (TSI) was used to analyze the stability of the emulsion, where the smaller the TSI value over a certain period of time, the more stable the emulsion.

Third, emulsions with different oil-to-water ratios were prepared. The demulsification voltages of the emulsion samples of different TQ-1 concentrations were determined using an electrical stability tester (Model 23C, FANN, Houston, TX, USA). The test temperature was set to 25 °C.

Fourth, emulsions having an oil-water ratio of 80:20 with TQ-1 concentrations of 0%, 1%, 2%, 3%, and 5% were formulated. The emulsions underwent shear emulsification at 2000 rpm using a high-speed shear emulsification mixer and then left to stand for 8 h. A focused beam reflectance measurement (FBRM) instrument was used to test the particle size distribution of each sample.

### 4.5. Evaluation of Rheological Performance

Viscosity vs. shear rate test: the viscosity at different shear rates of the emulsions with different TQ-1 concentrations was measured using a Physica MCR301 rheometer (Anton Paar, Vienna, Austria). The experiment used a plate rotor with a slit spacing of 0.047 mm. The shear rate ranged from 0.05 to 400 s^−1^, and the test temperature was set to 25 °C.

Three interval thixotropy test: the Physica MCR301 rheometer was used to simulate a “stationary-destroying-stationary” three-interval thixotropic test on the emulsions with different TQ-1 concentrations. The samples were sheared at a low speed at 5 s^−1^, then the shear rate was adjusted to 170 s^−1^. Finally, the shear rate was restored to 5 s^−1^. For each interval, the shearing time was 3 min. The test temperature was 25 °C. 

### 4.6. Performance of TQ-1 in OBDFs

#### 4.6.1. Preparation of OBDFs

Three different OBDFs were formulated according to Table 2. Formulation #1 is the conventional OBDF, Formulation #2 contained additional 0.3% TQ-1 in comparison with Formulation #1, and Formulation #3 contained additional 0.6% TQ-1 in comparison with Formulation #1. 

#### 4.6.2. Performance Evaluation of the OBDFs

The OBDFs were aged at a high-temperature rolling condition of 180 °C for 16 h using a high-temperature roller heating furnace (GW300-PLC, Qingdao Tongchun, Qingdao, China). The rheological parameters and demulsification voltages of the different OBDFs were measured using a six-speed rotary viscometer (ZNN-D6, Qingdao Tongchun, China) and an electrical stability meter (Model 23C, FANN, USA) at a temperature of 65 °C.

A HTHP static fluid loss tester (GGS42-2, Qingdao Tongchun, China) was used to measure the HTHP filtration losses of the aged OBDFs, where the test conditions were 180 °C at 3.5 MPa. 

After pouring the drilling fluid into the aging cell and allowing it to stand at room temperature for a period of time, the density of the upper half of the drilling fluid ρ_top_ and the density of the lower half of the drilling fluid ρ_bottom_ were measured, and the static sedimentation factor (SF) of the drilling fluid was calculated as follows:

(1) $$\mathrm{SF}=\frac{\rho_{\mathrm{bottom}}}{\rho_{\mathrm{top}}+\rho_{\mathrm{bottom}}}\times 100\%$$

where SF is the sedimentation factor (dimensionless), ρ_top_ is the density of the upper part of the drilling fluid column (g/cm^3^), and ρ_bottom_ is the bottom density of the drilling fluid column (g/cm^3^).

## Figures and Tables

**Table 1:** The effect of TQ-1 on the performance of OBDFs.

Type	Density (g/cm^3^)	AV (mPa·s)	PV (mPa·s)	YP (Pa)	YP/PV (Pa/(mPa·s))	G′/G″ (Pa/Pa)	FL_HTHP_ (mL)	ES (V)
1#	2.0	52.5	52	0.5	0.01	1.5/2.0	5.1	782
2#	2.0	59	53	6.0	0.11	3.5/5.5	4.8	1126
3#	2.0	63	54	9.0	0.16	4.0/6.5	4.7	1347

Aging conditions were 180 °C × 16 h; the high-pressure, high-temperature filtration loss experiments were carried out at 180 °C × 3.5 MPa.

**Table 2:** Preparation method of the OBDFs.

Order	Additives	Formulation # 1	Formulation # 2	Formulation # 3	Stirring Speed (r/min)	Stirring Time (min)
1	White oil	255 mL	255 mL	255 mL	/	/
2	Main emulsifier	10.5 g	10.5 g	10.5 g	5000	10
3	Secondary emulsifier	4.5 g	4.5 g	4.5 g	5000	10
4	20% CaCl_2_	45 mL	45 mL	45 mL	5000	20
5	CaO	9 g	9 g	9 g	5000	20
6	Organoclay	6 g	3 g	3 g	5000	20
7	Blown asphalt	12 g	12 g	12 g	5000	20
8	TQ-1	0 g	0.9 g	1.8 g	5000	20
9	Barite	660 g	660 g	660 g	5000	20

## Data Availability

Data is contained within the article.

## Appendix A

**Table A1:** List of abbreviations.

Abbreviations	Description
OBDFs	oil based drilling fluids
TQ -1	polyamide wax
FTIR	fourier transform infrared spectroscopy
TGA	thermogravimetric analysis
TSI	emulsion stability index
SF	sedimentation factor
HTHP	high-temperature and high-pressure
ZnO-NPs	nano-zinc oxide
nano-CaCO_3_	nano-calcium carbonate
PSBR	polystyrene-butadiene rubber
YP	yield point
AV	apparent viscosity
PV	plastic viscosity
ES	electrical stability
FBRM	focused beam reflectance measurement
